# Understanding Telemedicine's “New Normal”: Variations in Telemedicine Use by Specialty Line and Patient Demographics

**DOI:** 10.1089/tmj.2021.0041

**Published:** 2022-01-06

**Authors:** Connor Drake, Tyler Lian, Blake Cameron, Kate Medynskaya, Hayden B. Bosworth, Kevin Shah

**Affiliations:** ^1^ Department of Population Health Sciences, Duke University School of Medicine, Durham, North Carolina, USA.; ^2^ Department of Medicine, Duke University School of Medicine, Durham, North Carolina, USA.; ^3^ Private Diagnostic Clinic, Durham, North Carolina, USA.; ^4^ Center of Innovation to Accelerate Discovery and Practice Transformation (ADAPT), Durham Veterans Affairs Medical Center, Durham, North Carolina, USA.; ^5^ Duke University Health System, Durham, North Carolina, USA.

**Keywords:** telemedicine, telehealth, COVID-19, implementation, health equity

## Abstract

***Background:*** *Our objective was to examine the variation in telemedicine adoption by specialty line and patient demographic characteristics after the initial peak period of the coronavirus disease 2019 pandemic when in-person visits had resumed and visit volume returned to prepandemic levels.*

***Materials and Methods:*** *Aggregated encounter data were extracted for six service lines (dermatology, psychiatry, endocrinology, cardiology, orthopedics, and nonurgent primary care) in an integrated health system across three time periods: July 1 to September 30, 2019 (*n* = 239,803), July 1 to September 30, 2020 (*n* = 245,648), and December 29, 2019 to October 3, 2020 (*n* = 624,886). Risk ratios were calculated to assess the relative use of telemedicine compared with in-person encounters and telemedicine modality (i.e., synchronous audio/video vs. audio-only telephone) by patient race, age, sex, and insurance type.*

***Results:*** *By June 2020, total visit volume returned to prepandemic levels. Differences in patient demographics between July 1 to September 30, 2020 and the previous year's baseline were negligible. Telemedicine adoption varied by medical specialty, from 3.2% (dermatology) to 98.3% (psychiatry) of visits. African American and male patients were less likely to use telemedicine (telephone or video) compared with white and female patients. Among telemedicine encounters, African American, publicly insured, and older patients were less likely to use video compared with white, commercially insured, and younger patients.*

***Discussion:*** *Variation in telemedicine adoption and modality underscores the importance of balancing patient- and clinic-level implementation factors to promote sustainable, equitable telemedicine integration.*

***Conclusion:*** *Understanding current trends in the “new normal” of telemedicine provides valuable insights into future implementation and financing.*

## Introduction

In the months after the initial outbreak of the coronavirus disease 2019 (COVID-19) pandemic, telemedicine has become a new fixture of clinical outpatient care. The term “telemedicine” refers to a range of technologies that facilitate remote medical care through a two-way, synchronous communication between a patient and a physician or care team member.^1^ For years, practitioners and researchers have advocated for telemedicine to complement—and even supersede—in-person care as an efficient, convenient, and accessible alternative, especially for chronic care delivery and to improve access among rural patients.^2^ The pandemic compelled states, payers, and providers to overcome the long-standing implementation and financial barriers to telemedicine adoption.^3^ COVID-19 stimulated the establishment of a “new normal” for virtual, synchronous care that could become a standard of care moving forward.^4^

Although telemedicine has been critical for maintaining continuity of care in the midst of public health precautions, concerning trends related to equity and access have emerged, including disparities in race, age, and geography.^5–7^ The widening of this digital divide has been driven by social determinants of health, including lack of access to the internet or internet-enabled devices, housing insecurity, digital literacy, medical or technological mistrust, and issues related to health care access and reimbursement.^8^

The quality of telemedicine services is also dependent on a number of clinical considerations that are likely to vary by medical specialty, including patient condition and needs, the nature of necessary activities during a visit (e.g., physical examinations), patient and provider preferences, and reimbrusement.^9^ Despite a growing evidence base,^10–12^ comparative effectiveness research of telemedicine compared with in-person encounters for different specialty lines and services has, in general, been limited with the greatest evidence base being related to metabolic and cardiovascular disorders.^13,14^

As a result, understanding variations in the use of telemedicine, both by different clinical specialty lines and by different patient groups, is critical to informing future priorities for telemedicine research and reimbursement policies. The latter has and continues to be a driver of telemedicine implementation and adoption.^15,16^ In immediate response to the pandemic, both public and private payers have implemented reimbursement parity for a range of synchronous video and audio-only (e.g., telephone) services relative to in-person visits.^17^ Already, however, some payers have reduced reimbursement for audio-only visits.^18^ Changes in telemedicine reimbursement policies may have important and disproportionate ramifications for certain service lines and vulnerable patient populations in telemedicine's “new normal,” as health care systems continue to assess the financial implications of virtual care.

In this descriptive study, our objective was to examine variation in telemedicine adoption by specialty line and patient demographic characteristics in the months after the initial peak period of the pandemic, using aggregated encounter data at a large integrated health system in North Carolina. The present study makes two novel contributions to the burgeoning telemedicine literature in the time of the COVID-19 pandemic. First, we compared longitudinal trends in telemedicine adoption between six different clinical service lines (dermatology, psychiatry, endocrinology, cardiology, orthopedics, and nonurgent primary care) to assess specialty-specific differences. Second, we chose the time period of this study (July 1 to September 30, 2020) to investigate the stable operation of telemedicine, when the health care system began to transition out of emergency protocols, clinics reopened to in-person visits, and patient volumes returned to prepandemic levels. This time period may better represent a long-term view of the “new normal” of telemedicine, as opposed to its accelerated adoption in the face of a public health crisis.

## Materials and Methods

### DATA

In response to the COVID-19 pandemic, during the week of March 16, 2020, the Duke Health system expanded telemedicine services across all outpatient clinical services to support a diverse patient population from across the state in Durham, North Carolina. On March 10, Governor Roy Cooper had declared a state of emergency with additional travel, gathering, and business restrictions in the following weeks.^19^ By October 2020, North Carolina had the seventh highest number of confirmed cases in the country.^20^ To facilitate the transition to telemedicine, departments were provided organizational support (e.g., technical assistance, training, scheduling, documentation, and administrative support) to conduct visits through either telephone call or audio-video technology integrated in the electronic health record (Epic, Verona, WI; Zoom, San Jose, CA), based on patient, provider, or clinic preferences. Institutional considerations and strategies for telemedicine implementation are described elsewhere.^21^

We queried an internal data dashboard to abstract data on all completed outpatient visits within six clinical service lines: dermatology, psychiatry, endocrinology, cardiology, orthopedics, and primary care (excluding urgent care). These medical specialties were chosen to represent a diversity of clinical conditions and patient populations, procedural emphases, representativeness, and volume. Clinical encounters were classified as one of three visit modalities: in-person, telephone, or video, with the latter two constituting “telemedicine.” The generated report also included aggregated information about four patient demographics: race, payer (i.e., primary insurance), age, and sex.

This study considered retrospective cohorts from three main time periods of interest: July 1 to September 30, 2020 (*n* = 245,648), as the primary study period; July 1 to September 30, 2019 (*n* = 239,803), as an analogous pre-COVID baseline; and December 29, 2019 to October 3, 2020 (*n* = 624,886), to understand the longitudinal trends of telemedicine adoption over the course of the pandemic.

This study was reviewed by the Duke University Health System (DUHS) Institutional Review Board and was granted an exemption.

### DATA ANALYSIS

First, we compared the distribution of patient demographic characteristics between July 1 and September 30, 2020, and an analogous baseline period (July 1 to September 30, 2019), using Pearson's chi-squared tests, to understand possible changes in patient composition due to the pandemic. Effect sizes of the chi-squared test are quantified by the bias-corrected Cramér's V, which ranges from 0 (no association between the two nominal variables) to 1 (complete association). In this study, *V* < 0.1 was considered a small effect size.^22^

Second, to evaluate trends, outliers, and shifts in telemedicine usage over the course of the pandemic, we plotted the weekly volume of in-person, video, and telephone visits by medical specialty from December 29, 2019 to October 3, 2020.

Finally, for visits that occurred between July 1 and September 30, 2020, we calculated unadjusted risk ratios (RRs) by medical specialty to assess both the relative use of telemedicine (i.e., vs. in-person) and the relative use of modes within telemedicine (i.e., video vs. telephone) by patient race, payer, age, and sex, using the largest group in each demographic category as the reference group. In this study, statistical significance was defined as *p*-value <0.05, and 95% confidence intervals (CIs) were reported.

## Results

### DISTRIBUTION OF PATIENT DEMOGRAPHICS

Patient demographic characteristics, aggregated across the six queried medical specialties, are provided in *Table 1*. From July to September 2020, the majority of patients identified as white (63.1%), and about one-fourth of patients identified as black/African American (25.8%). The three largest insurance types were commercial (51.9%), Medicare (37.2%), and Medicaid (5.8%). About 15.4% of patients were 75 years old or older, and only 2.5% of patients were <10 years old. More patients identified as female (60.0%) than male (40.0%). See Supplementary Appendix SA1 for distributions of patient demographics by specialty.

**Table 1.** Description of and Comparison between Patient Characteristics, July 1 to September 30, 2019 and 2020, Aggregated Across Six Clinical Service Lines

DEMOGRAPHIC, % (*n*)	JULY–SEPTEMBER 2019	JULY–SEPTEMBER 2020	*p*	V^a^
***N***	239,803	245,648		
Race or ethnicity				
White	64.8 (155,363)	63.1 (155,043)	<0.001^*^	0.0178
Black/African American	24.6 (58,878)	25.8 (63,478)		
Hispanic	3.9 (9,428)	3.9 (9,643)		
Unknown	3.1 (7,501)	3.3 (8,143)		
Asian	2.0 (4,829)	2.1 (5,111)		
Multiracial	1.2 (2,980)	1.3 (3,256)		
American Indian or Alaskan Native	0.3 (648)	0.3 (745)		
Native Hawaiian or Other Pacific Islander	0.1 (176)	0.1 (188)		
Payer				
Commercial	52.3 (125,349)	51.9 (127,533)	0.894	0.0001
Medicare	36.5 (87,603)	37.2 (91,423)		
Medicaid	5.7 (13,775)	5.8 (14,351)		
Self-pay	4.0 (9,498)	3.5 (8,584)		
Miscellaneous	1.5 (3,578)	1.5 (3,757)		
Age at encounter, years				
Ages 75+ (silent and greatest)	15.9 (38,091)	15.4 (37,915)	<0.001^*^	0.0353
Ages 56–74 (baby boomers)	35.6 (85,451)	36.9 (90,742)		
Ages 40–55 (Gen X)	22.3 (53,501)	23.1 (56,753)		
Ages 25–39 (Millennials)	14.5 (34,880)	14.9 (36,657)		
Ages 10–24 (Gen Z)	8.8 (21,222)	7.1 (17,527)		
Ages 0–9 (Gen Alpha)	2.8 (6,658)	2.5 (6,054)		
Sex				
Female	59.3 (142,312)	60.0 (147,415)	<0.001^*^	0.0070
Male	40.6 (97,459)	40.0 (98,214)		
Unknown	<0.1 (32)	<0.1 (19)		

Results are aggregated over six clinical service lines: dermatology, psychiatry, cardiology, endocrinology, orthopedics, and nonurgent primary care. Demographics by specialty line are presented in Supplementary Appendix SA1.

^*^ *p* < 0.05.

^a^ Effect sizes of Pearson's chi-squared test are quantified by the bias-corrected Cramér's *V*. Here, *V* < 0.1 is considered a small effect size.

Comparing the 3-month period from July to September, there were no notable differences in either overall patient volume or overall demographic distribution between 2020 and the previous year's baseline based on the bias-corrected Cramér's V value (*Table 1*). Within each medical specialty, demographic distributions and patient volumes also remained stable, with a few exceptions highlighted in Supplementary Appendix SA1.

From July to September 2019, 99.97% of visits among the six queried service lines were in-person. In contrast, from July to September 2020, 76.99% of visits were in-person, 11.53% were over video, and 11.48% were over the phone. In this time period, psychiatry reported the highest levels of telemedicine usage (98.3% of visits), followed by endocrinology (64.9%), nonurgent primary care (20.8%), cardiology (10.0%), orthopedics (4.7%), and dermatology (3.2%).

### USE OF TELEMEDICINE OVER TIME

**Fig. 1.**
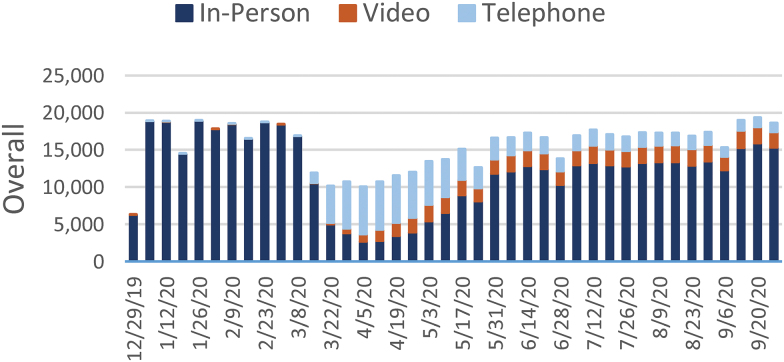
Weekly overall visit volume from December 29, 2019, to October 3, 2020. Stacked bars indicate the visit modality: in-person (dark blue), video (orange), or telephone (light blue). “Overall” refers to the aggregation of visits across the six service lines of this study: dermatology, psychiatry, endocrinology, cardiology, orthopedics, and nonurgent primary care. Color images are available online.

**Fig. 2.**
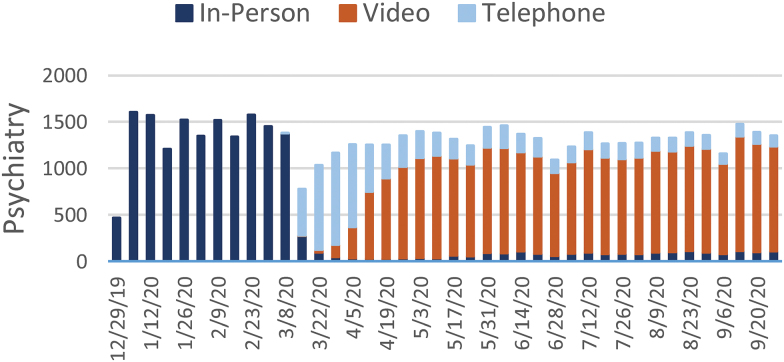
Weekly visit volume in psychiatry from December 29, 2019, to October 3, 2020. Stacked bars indicate the visit modality: in-person (dark blue), video (orange), or telephone (light blue). Color images are available online.

**Fig. 3.**
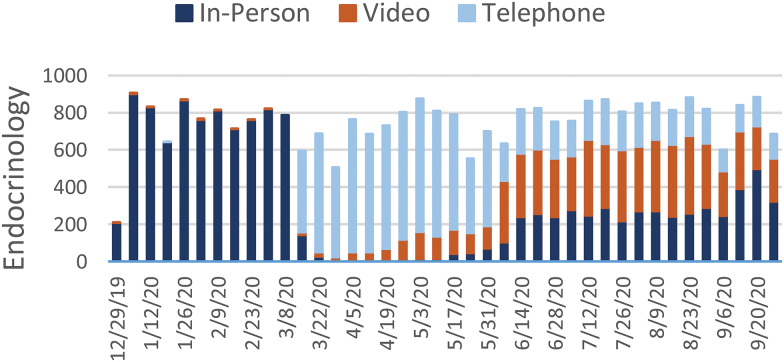
Weekly visit volume in endocrinology from December 29, 2019, to October 3, 2020. Stacked bars indicate the visit modality: in-person (dark blue), video (orange), or telephone (light blue). Color images are available online.

**Fig. 4.**
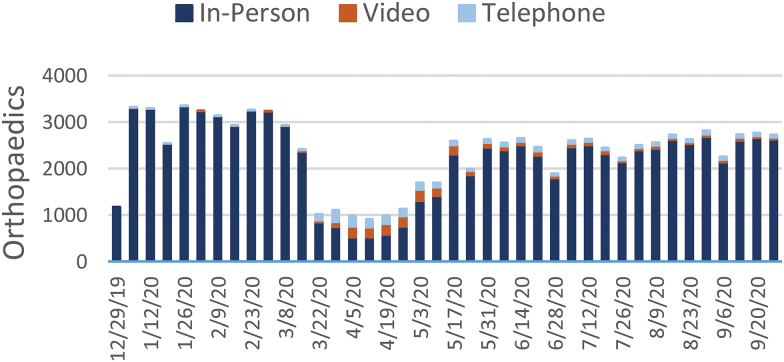
Weekly visit volume in orthopedics from December 29, 2019, to October 3, 2020. Stacked bars indicate the visit modality: in-person (dark blue), video (orange), or telephone (light blue). Color images are available online.

**Fig. 5.**
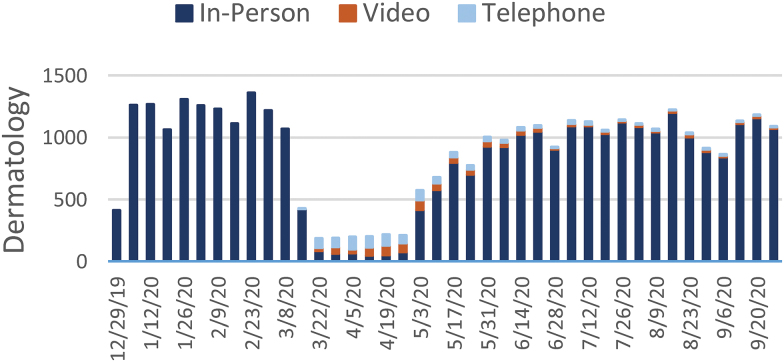
Weekly visit volume in dermatology from December 29, 2019, to October 3, 2020. Stacked bars indicate the visit modality: in-person (dark blue), video (orange), or telephone (light blue). Color images are available online.

**Fig. 6.**
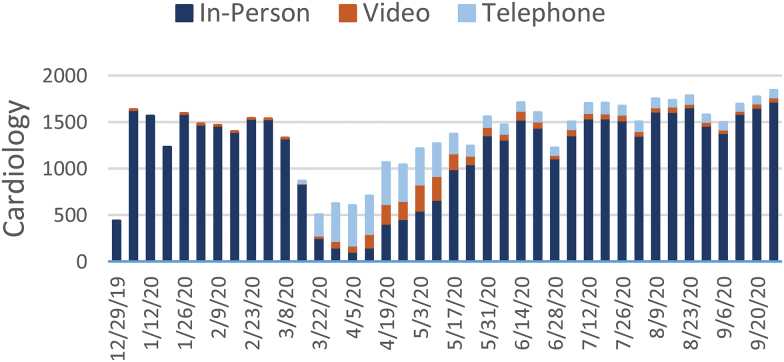
Weekly visit volume in cardiology from December 29, 2019, to October 3, 2020. Stacked bars indicate the visit modality: in-person (dark blue), video (orange), or telephone (light blue). Color images are available online.

**Fig. 7.**
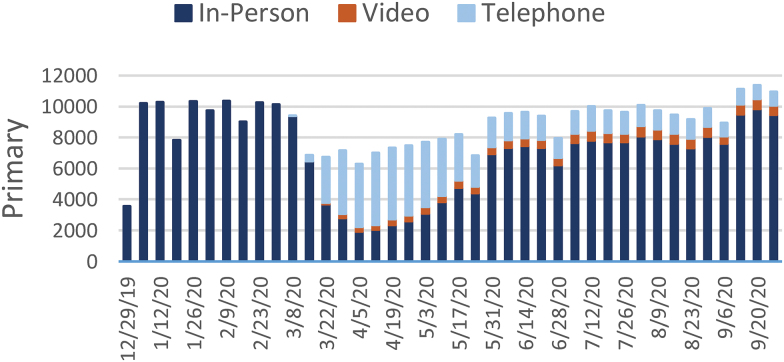
Weekly visit volume in nonurgent primary care from December 29, 2019, to October 3, 2020. Stacked bars indicate the visit modality: in-person (dark blue), video (orange), or telephone (light blue). Color images are available online.

*Figures 1–7* report the weekly volume of in-person, video, and telephone visits from January to September 2020. In mid-March, when the health system implemented a system-wide policy to restrict in-person visits due to the pandemic, clinics began a partial transition to telemedicine. By June, visit volumes in all six service lines had returned to prepandemic levels.

The adoption of video and telephone visits varied by medical specialty, both in terms of rate and relative use. Psychiatry (*Fig. 2*) and endocrinology (*Fig. 3*) specialty lines experienced almost no decrease in visit volume, and these specialties' use of telemedicine persisted to the end of the study period. On the contrary, orthopedics (*Fig. 4*) and dermatology (*Fig. 5*) clinics did not utilize video or telephonic telemedicine technologies to maintain prepandemic encounter volume, electing to resume in-person visits gradually over the course of May and June. Both cardiology (*Fig. 6*) and nonurgent primary care (*Fig. 7*) saw reductions in encounter volume in April, partially supplemented by telephonic encounters until in-person volume returned to prepandemic levels.

### RELATIVE USE OF TELEMEDICINE

*Table 2* reports the relative use of telemedicine (i.e., either telephone or video) by race, payer, age, and sex from July to September 2020, aggregated across the six clinical service lines. See Supplementary Appendix SB2 for more details by specialty. RRs larger than 1 indicate that the patients of that demographic group used the indicated visit modality more often than patients in the reference group.

**Table 2.** Relative Use of Telemedicine and Relative Use of Video, Within Telemedicine, by Patient Demographics, Aggregated Across Six Clinical Service Lines, July 1 to September 30, 2020

	USE OF TELEMEDICINE^a^		USE OF VIDEO, WITHIN TELEMEDICINE SUBGROUP^a^	
	RR	95% CI	RR	95% CI
Race or ethnicity	Reference group: White			
Black/African American	0.895^*^	0.879–0.910	0.587^*^	0.571–0.602
Hispanic	0.803^*^	0.770–0.837	1.187^*^	1.147–1.229
Asian	0.948^*^	0.910–0.988	1.081^*^	1.040–1.124
Multiracial	1.152^*^	1.089–1.219	1.207^*^	1.151–1.266
American Indian or Alaskan Native	0.917	0.800–1.050	0.849	0.720–1.001
Native Hawaiian or Other Pacific Islander	0.936	0.717–1.222	0.824	0.591–1.149
Payer	Reference group: Commercial			
Medicare	1.023^*^	1.007–1.039	0.770^*^	0.756–0.785
Medicaid	1.053^*^	1.021–1.086	0.729^*^	0.699–0.760
Self-pay	1.290^*^	1.247–1.336	0.705^*^	0.671–0.741
Age at encounter, years	Reference group: Ages 56–74 (baby boomers)			
Ages 75+ (silent, greatest)	1.110^*^	1.085–1.134	0.969^*^	0.942–0.997
Ages 40–55 (Gen X)	1.180^*^	1.158–1.203	1.207^*^	1.181–1.234
Ages 25–39 (Millennials)	1.329^*^	1.301–1.356	1.255^*^	1.226–1.284
Ages 10–24 (Gen Z)	0.908^*^	0.878–0.938	1.332^*^	1.290–1.375
Ages 0–9 (Gen Alpha)	0.695^*^	0.654–0.740	1.286^*^	1.213–1.364
Sex	Reference group: Female			
Male	0.779^*^	0.767–0.791	1.027^*^	1.010–1.045

Results are aggregated over six medical specialties: dermatology, psychiatry, cardiology, endocrinology, orthopedics, and nonurgent primary care. Results by specialty line are presented in Supplementary Appendix SB2.

^*^ *p* < 0.05.

^a^ Visits were classified as one of three modalities: in-person, telephone, and video, with the latter two constituting “telemedicine.”

CI, confidence interval; RR, risk ratio.

Black/African American patients were 0.895 (CI 0.879–0.910) times less likely than white patients to use telemedicine for their visit. Both Hispanic patients (RR 0.803, CI 0.770–0.837) and Asian patients (RR 0.948, CI 0.910–0.988) were also less likely to use telemedicine compared with white patients.

Medicare patients (RR 1.023, CI 1.007–1.039) and Medicaid patients (RR 1.053, CI 1.021–1.086) were both only slightly more likely to use telemedicine than commercially insured patients. Patients who self-paid for their visit were more likely (RR 1.290, CI 1.247–1.336) to use telemedicine as well, compared with commercially insured patients.

Compared with patients in the “baby boomer” generation (ages 56–74 years), millennial patients (ages 25–39 years) were the most likely (RR 1.329, CI 1.301–1.356) to use telemedicine, followed by Gen X patients (ages 40–55 years; RR 1.180, CI 1.158–1.203) and patients in the “silent” or “greatest” generations (ages 75+ years; RR 1.110, CI 1.085–1.134). In contrast, patients in Gen Z (ages 10–24 years; RR 0.908, CI 0.878–0.938) and Gen Alpha (ages 0–9 years; RR 0.695, CI 0.654–0.740) were both less likely to use telemedicine compared with baby boomers.

Finally, male patients were 0.779 (CI 0.767–0.791) times less likely to use telemedicine than female patients.

### RELATIVE USE OF VIDEO WITHIN TELEMEDICINE

*Table 2* also reports the relative use of video within the subgroup of visits that were classified as telemedicine (i.e., either telephone or video) by race, payer, sex, and age. Again, Supplementary Appendix SB2 has more details by specialty.

Among the patients who used telemedicine, black/African American patients were much less likely (RR 0.587, CI 0.571–0.602) to use video for their visit compared with white patients. Hispanic patients (RR 1.187, CI 1.147–1.229) and Asian patients (RR 1.081, CI 1.040–1.124) were both more likely than white patients to use video, even though they—like black/African American patients—were less likely to use telemedicine overall.

Medicare patients (RR 0.770, CI 0.756–0.785), Medicaid patients (RR 0.729, CI 0.699–0.760), and self-pay patients (RR 0.705, CI 0.671–0.741) were all less likely than commercially insured patients to use video, even though all three groups were slightly more likely to use telemedicine overall.

All age demographic groups younger than baby boomers were more likely to use video compared with baby boomers (ages 56–74 years). Male patients were only slightly more likely (RR 1.027, CI 1.010–1.045) to use video than female patients.

## Discussion

In less than a year, the COVID-19 pandemic has made a lasting, transformational mark on American health care systems, including the rapid expansion of telemedicine to deliver routine outpatient care. By directing attention to the months after the initial peak period of the pandemic, the present study extends the previous telemedicine literature to better understand this “new normal” of health care delivery, including trends of adoption both by clinical specialty line and patient demographic groups. In our main study period, severe emergency protocols had been lifted, outpatient clinics returned to treating patients in person Oxford, and visit volumes returned to prepandemic volumes without major changes in patient demographic composition, allowing our study to reveal insights about the use of telemedicine outside of the initial acute crisis period.

Our study is one of the first to compare the adoption of telemedicine across several clinical service lines. From July to September 2020, we found that the use of telemedicine (i.e., either telephone or video) varied widely across medical specialty service lines, despite existing evidence bases of telemedicine across these service lines.^14,23–32^ These patterns of differential uptake may underscore important clinical and implementation considerations that should be further investigated and described.

These differences in adoption at a department-level may be clarified by understanding the clinic-specific barriers and facilitators of telemedicine (e.g., leadership engagement, level of technical assistance, provider preferences) and the specific encounter types that best lend themselves to telemedicine technologies (e.g., certain clinical examinations, interactions, procedures, and/or other clinical processes). Additional research should focus on rigorous comparative effectiveness trials to identify telemedicine applications across medical specialties that are feasible, accessible, and effective in comparison with traditional in-person care. These findings may also be related to the important and context-specific implementation determinants of telemedicine adoption.^33^

Our findings also report significant variations in telemedicine use by patient race, age, sex, and insurance type, aligning with previous literature that calls attention to ongoing and exacerbated inequities associated with telemedicine expansion.^5,7,34,35^ Aggregating across six service lines, we found that black/African American patients and Hispanic patients were less likely than white patients to use telemedicine for their visit. Male patients were also less likely to use telemedicine than female patients. Among patients who used telemedicine, black/African American patients were less likely to use video for their visit than white patients, and both Medicare patients and Medicaid patients were less likely to use video than commercially insured patients. Younger patients were more likely to use video than patients in the baby boomer generation and older. These variations in use could be due to the underlying differences in various social drivers, patient and provider preferences, technological literacy, access to a reliable internet connection, and patient condition complexity and medical visit type (e.g., evaluation, diagnostic, consultation, or intervention).^7,36–38^

These findings are consistent with the existing literature, which largely suggests that being younger, white, higher income, and female are associated with higher utilization of telemedicine.^34,35,39,40^ However, these patterns appear to vary by institution and patient population.^41,42^ Differing patterns and associations, however, are not surprising given the variations in study setting, telemedicine intervention, patient population, specialty line, and data analysis plan among telemedicine studies, which makes comparing single-institution studies challenging. Regardless, it is clear that system-wide implementations of telemedicine must focus explicit and proactive attention to policies that address the social and economic structures that shape health and access to care, such as racism, resource distribution, and education access.^5–7^

Our results have implications for policymakers who are considering the costs and benefits of reimbursing telemedicine encounters at parity with in-person encounters, which has been long understood as a mechanism for promoting adoption.^43^ In the past few months, several payers have proposed to decrease or eliminate reimbursement for telephone encounters compared with video or in-person visits.^18^ The findings of this study suggest that this may result in restricted health care access for certain vulnerable communities, including black/African American patients; Medicare, Medicaid, and self-pay patients; and patients older than 75 years.

This study should be interpreted in the context of several limitations. First, data were collected from a single integrated academic health system, which may limit generalizability to other health systems, practice types, geographic areas, and public insurers; however, while the Duke Health system primarily resides in a populated metropolitan area, it has a large primary care network and specialty referral base from the surrounding rural areas and overall serves a racially, geographically, and economically diverse population. Second, only unadjusted differences in relative telemedicine use could be calculated. As such, our analysis is descriptive in nature, and we caution against causal interpretation. Future research should expand upon existing research about patient characteristics associated with telemedicine use during the COVID-19 pandemic^34,35,41^ to include factors such as clinical complexity, socioeconomic status, and other demographics. Finally, our analysis was limited to a time period up to September 30, 2020, when the effects of the pandemic may still influence which patients seek care and use telemedicine.

Despite these limitations, our study complements existing research by taking a step toward understanding the postpandemic future of telemedicine, revealing variations in telemedicine use by both clinical specialty line and patient demographics. When taken together, our results suggest that there is no “one-size-fits-all” approach to telemedicine adoption, instead underscoring the need to tailor strategies of telemedicine implementation to clinical service lines and other considerations of patient barriers to care. Existing evidence suggests that leadership engagement, resource availability, clinician design involvement, and workforce development are critical drivers of implementation.^44–46^ Health systems and policymakers should leverage lessons learned during the COVID-19 pandemic to inform the sustainable adoption of telemedicine. The effective, efficient, and equitable implementation of telemedicine in the “new normal” will require attention to clinical and organizational implementation factors alongside structural disparities that may affect patients' access to care.

## Conclusion

Using aggregate encounter data, we examined variation in telemedicine use by medical specialty (dermatology, psychiatry, endocrinology, cardiology, orthopedics, and nonurgent primary care) and demographic groups (race, age, sex, and insurance type) after the peak period of the pandemic when visit volume returned to prepandemic levels. There is significant heterogeneity in telemedicine use by medical specialty service line and across patient groups. This variation raises important issues related to both patient- and clinic-level implementation factors to promote sustainable, equitable telemedicine integration. By understanding the “new normal” of telemedicine these findings provide insights to the long-term sustainability of telemedicine that are relevant to both policymakers and practitioners.

## Supplementary Material

Supplemental data

Supplemental data

## Supplementary Material

Supplementary Appendix SA1

Supplementary Appendix SB2
