# Supplementary material for: Understanding Telemedicine's “New Normal”: Variations in Telemedicine Use by Specialty Line and Patient Demographics
Source: Telemed J E Health. 2022 Jan 6;28(1):51–9. doi: 10.1089/tmj.2021.0041 (PMC8785715; doi:10.1089/tmj.2021.0041)
Supplement: Supplemental data [file Supp_AppendixSA1.docx]

**Appendix A**

**Figure A1. Distribution of Patient Race by Clinical Service Line.** The distribution of patient race during COVID-19 (July to September 2020) remained similar compared to the baseline period (July to September 2019), both within each specialty and aggregated across all specialties. Numbers on the right-hand side represent the total visit volume for the respective time period. Derm = Dermatology; Psy = Psychiatry; Endo = Endocrinology; Card = Cardiology; Orth = Orthopaedics; Pri = Non-urgent primary care; TOT = Total over the six medical specialties.


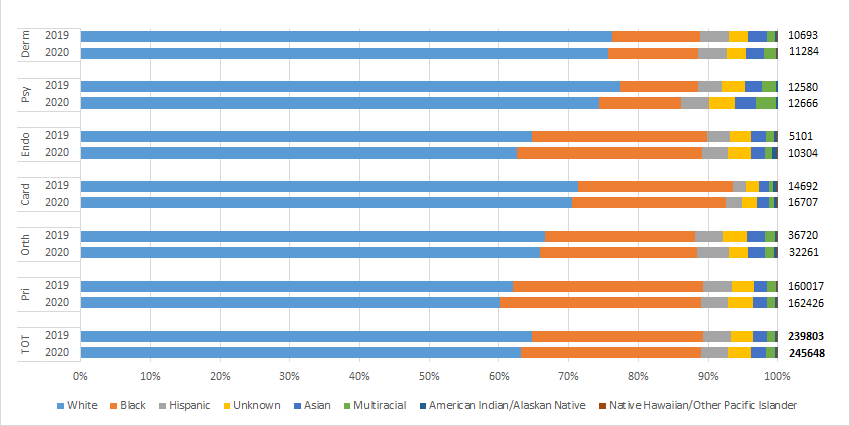


**Figure A2. Distribution of Visit Payer by Clinical Service Line.** The distribution of patient payer during COVID-19 (July to September 2020) remained similar compared to the baseline period (July to September 2019), both within each specialty and aggregated across all specialties. Notable exceptions included the increase, within psychiatry, of the proportion of Medicare patients from 15% to 42% and the subsequent decrease, also within psychiatry, of the proportion of patients on commercial plans (64% to 42%). Numbers on the right-hand side represent the total visit volume for the respective time period. Derm = Dermatology; Psy = Psychiatry; Endo = Endocrinology; Card = Cardiology; Orth = Orthopaedics; Pri = Non-urgent primary care; TOT = Total over the six medical specialties.


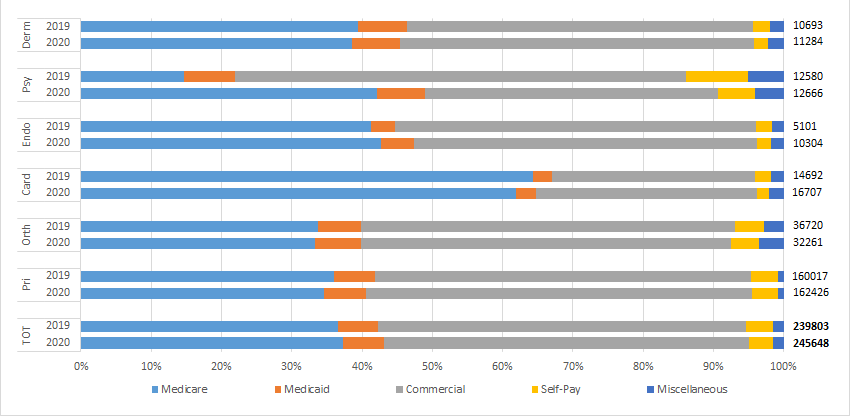


**Figure A3. Distribution of Patient Age by Clinical Service Line.** The distribution of patient age during COVID-19 (July to September 2020) remained similar compared to the baseline period (July to September 2019), both within each specialty and aggregated across all specialties. Notable exceptions included the increase, within psychiatry, of the proportion of patients 75 and older (4% to 23%) and the subsequent decrease, also within psychiatry, of the proportion of patients between 10 and 24 (27% to 6%). Numbers on the right-hand side represent the total visit volume for the respective time period. Derm = Dermatology; Psy = Psychiatry; Endo = Endocrinology; Card = Cardiology; Orth = Orthopaedics; Pri = Non-urgent primary care; TOT = Total over the six medical specialties.


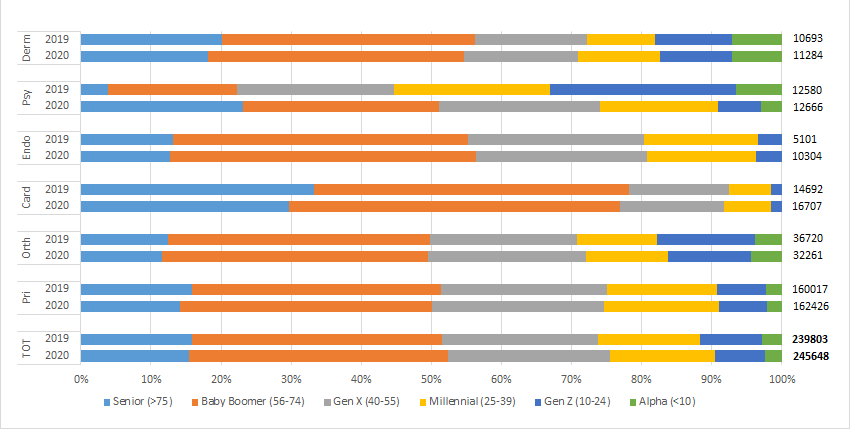


**Figure A4. Distribution of Patient Sex by Clinical Service Line.** The distribution of patient sex during COVID-19 (July to September 2020) remained similar compared to the baseline period (July to September 2019), both within each specialty and aggregated across all specialties. Numbers on the right-hand side represent the total visit volume for the respective time period. Derm = Dermatology; Psy = Psychiatry; Endo = Endocrinology; Card = Cardiology; Orth = Orthopaedics; Pri = Non-urgent primary care; TOT = Total over the six medical specialties.


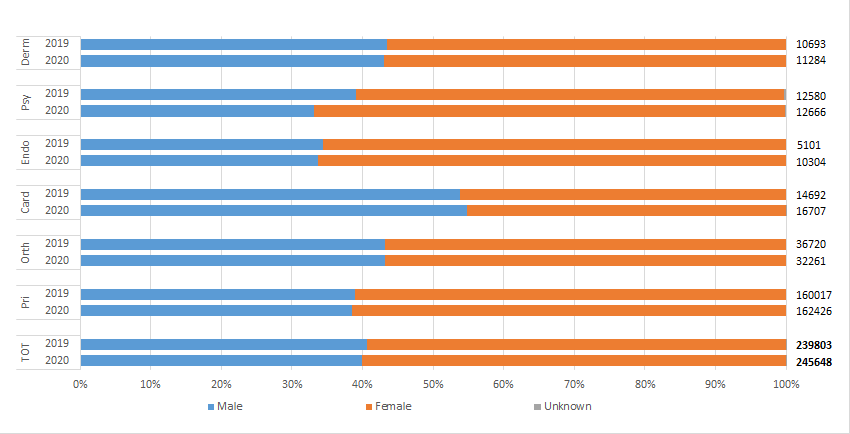


**Table A1. Comparison of the COVID-19 Distribution of Patient Demographics to Baseline, by Clinical Service Line.** The distribution of each demographic factor (race, payer, age, sex) during COVID-19 (July to September 2020) is compared to the baseline distribution (July to September 2019) using Pearson’s chi-squared test. “Overall” refers to the total distribution of the six medical specialties. Effect sizes of the chi-squared test are quantified by the bias-corrected Cramér’s V, which ranges from 0 (no association between the two nominal variables) to 1 (complete association). Here, Cramér’s V < 0.1 is considered a small effect size, and V > 0.5 is considered a large effect size (Cohen, J. 1988).

| **Distribution** | **Specialty** | ***P*** | **Effect Size (V)** |
| --- | --- | --- | --- |
| Race | Dermatology | 0.0798 | 0.0161 |
|  | Psychiatry | <0.0001 | 0.0396 |
|  | Endocrinology | 0.0586 | 0.0207 |
|  | Cardiology | 0.0312 | 0.0164 |
|  | Orthopaedics | <0.0001 | 0.0285 |
|  | Primary | <0.0001 | 0.0223 |
|  | **Overall** | **<0.0001** | **0.0178** |
| Payer | Dermatology | 0.0058 | 0.0219 |
|  | Psychiatry | <0.0001 | 0.3083 |
|  | Endocrinology | 0.0002 | 0.0343 |
|  | Cardiology | <0.0001 | 0.0351 |
|  | Orthopaedics | <0.0001 | 0.0212 |
|  | Primary | <0.0001 | 0.0160 |
|  | **Overall** | **<0.0001** | **0.0135** |
| Age | Dermatology | <0.0001 | 0.0367 |
|  | Psychiatry | <0.0001 | 0.3904 |
|  | Endocrinology | 0.2946 | 0.0078 |
|  | Cardiology | <0.0001 | 0.0362 |
|  | Orthopaedics | <0.0001 | 0.0368 |
|  | Primary | <0.0001 | 0.0264 |
|  | **Overall** | **<0.0001** | **0.0353** |
| Sex | Dermatology | 0.4670 | 0.0000 |
|  | Psychiatry | <0.0001 | 0.0626 |
|  | Endocrinology | 0.3684 | 0.0000 |
|  | Cardiology | 0.1712 | 0.0070 |
|  | Orthopaedics | 0.9648 | 0.0000 |
|  | Primary | 0.0117 | 0.0041 |
|  | **Overall** | **<0.0001** | **0.0070** |
