# Supplementary material for: Understanding Telemedicine's “New Normal”: Variations in Telemedicine Use by Specialty Line and Patient Demographics
Source: Telemed J E Health. 2022 Jan 6;28(1):51–9. doi: 10.1089/tmj.2021.0041 (PMC8785715; doi:10.1089/tmj.2021.0041)
Supplement: Supplemental data [file Supp_AppendixSB2.docx]

**Appendix B**

**Table B1. Relative Use of Telemedicine and Relative Use of Video Within Telemedicine by Patient Race, July 1 to September 30, 2020, by Clinical Service Line^a^**

| **vs. White** |  | **Use of Telemedicine** | | | **Use of Video, Within Telemedicine Subgroup** | | |
| --- | --- | --- | --- | --- | --- | --- | --- |
|  |  | **RR** | **CI LL** | **CI UL** | **RR** | **CI LL** | **CI UL** |
| Black | Dermatology | 2.107* | 1.650 | 2.691 | 0.542* | 0.398 | 0.738 |
|  | Psychiatry | 0.981* | 0.971 | 0.990 | 0.903* | 0.881 | 0.925 |
|  | Endocrinology | 1.029 | 0.997 | 1.063 | 0.722* | 0.688 | 0.757 |
|  | Cardiology | 0.988 | 0.884 | 1.105 | 0.710* | 0.595 | 0.849 |
|  | Orthopaedics | 0.660* | 0.578 | 0.755 | 0.645* | 0.538 | 0.774 |
|  | Primary | 0.951* | 0.930 | 0.971 | 0.568* | 0.554 | 0.582 |
|  | **Overall** | **0.895*** | **0.879** | **0.910** | **0.587*** | **0.571** | **0.602** |
| Hispanic | Dermatology | 0.947 | 0.534 | 1.680 | 0.646 | 0.328 | 1.270 |
|  | Psychiatry | 1.009* | 1.003 | 1.015 | 1.073* | 1.062 | 1.085 |
|  | Endocrinology | 0.975 | 0.901 | 1.055 | 0.919 | 0.837 | 1.008 |
|  | Cardiology | 0.874 | 0.631 | 1.210 | 1.040 | 0.691 | 1.563 |
|  | Orthopaedics | 0.648* | 0.492 | 0.854 | 1.021 | 0.783 | 1.330 |
|  | Primary | 0.725* | 0.684 | 0.768 | 0.433* | 0.408 | 0.459 |
|  | **Overall** | **0.803*** | **0.770** | **0.837** | **1.187*** | **1.147** | **1.229** |
| Asian | Dermatology | 0.963 | 0.480 | 1.930 | 1.357* | 1.026 | 1.793 |
|  | Psychiatry | 0.958* | 0.938 | 0.980 | 1.054* | 1.036 | 1.072 |
|  | Endocrinology | 0.963 | 0.884 | 1.049 | 1.117* | 1.041 | 1.198 |
|  | Cardiology | 0.961 | 0.695 | 1.329 | 1.411* | 1.038 | 1.917 |
|  | Orthopaedics | 0.732 | 0.524 | 1.023 | 0.851 | 0.580 | 1.247 |
|  | Primary | 0.897* | 0.850 | 0.947 | 1.142* | 1.062 | 1.228 |
|  | **Overall** | **0.948*** | **0.910** | **0.988** | **1.081*** | **1.040** | **1.124** |
| Multiracial | Dermatology | 1.231 | 0.555 | 2.732 | 1.292 | 0.892 | 1.871 |
|  | Psychiatry | 0.993 | 0.979 | 1.008 | 1.018 | 0.991 | 1.047 |
|  | Endocrinology | 1.017 | 0.889 | 1.162 | 0.928 | 0.790 | 1.091 |
|  | Cardiology | 1.132 | 0.690 | 1.860 | 1.443 | 0.912 | 2.283 |
|  | Orthopaedics | 0.910 | 0.597 | 1.386 | 1.285 | 0.945 | 1.748 |
|  | Primary | 0.970 | 0.891 | 1.056 | 1.206* | 1.080 | 1.346 |
|  | **Overall** | **1.152*** | **1.089** | **1.219** | **1.207*** | **1.151** | **1.266** |
| American Indian or Alaskan Native | Dermatology | 0.000 | - | - | - | - | - |
|  | Psychiatry | 0.932 | 0.830 | 1.046 | 1.039 | 0.952 | 1.134 |
|  | Endocrinology | 1.015 | 0.853 | 1.208 | 0.633* | 0.458 | 0.875 |
|  | Cardiology | 0.783 | 0.384 | 1.597 | 0.721 | 0.223 | 2.332 |
|  | Orthopaedics | 0.794 | 0.362 | 1.742 | 0.964 | 0.432 | 2.150 |
|  | Primary | 0.989 | 0.817 | 1.198 | 0.970 | 0.723 | 1.302 |
|  | **Overall** | **0.917** | **0.800** | **1.050** | **0.849** | **0.720** | **1.001** |
| Native Hawaiian or Pacific Islander | Dermatology | 0.000 | - | - | - | - | - |
|  | Psychiatry | 1.013* | 1.011 | 1.015 | 1.086* | 1.080 | 1.093 |
|  | Endocrinology | 1.124 | 0.782 | 1.614 | 1.218 | 0.937 | 1.584 |
|  | Cardiology | 1.244 | 0.199 | 7.785 | 2.525* | 2.353 | 2.708 |
|  | Orthopaedics | 0.000 | - | - | - | - | - |
|  | Primary | 1.094 | 0.808 | 1.481 | 0.868 | 0.519 | 1.451 |
|  | **Overall** | **0.936** | **0.717** | **1.222** | **0.824** | **0.591** | **1.149** |

RR = risk ratio, CI = confidence interval, LL = lower limit, UL = upper limit

^a^ “Overall” refers to the aggregation of the six medical specialties listed.

* = P < 0.05.

**Table B2. Relative Use of Telemedicine and Relative Use of Video Within Telemedicine by Patient Payer, July 1 to September 30, 2020, by Clinical Service Line^a^**

| **vs. Commercial** |  | **Use of Telemedicine** | | | **Use of Video, Within Telemedicine Subgroup** | | |
| --- | --- | --- | --- | --- | --- | --- | --- |
|  |  | **RR** | **CI LL** | **CI UL** | **RR** | **CI LL** | **CI UL** |
| Medicare | Dermatology | 0.745* | 0.597 | 0.928 | 0.769* | 0.617 | 0.959 |
|  | Psychiatry | 0.998 | 0.993 | 1.003 | 1.052* | 1.041 | 1.064 |
|  | Endocrinology | 0.958* | 0.930 | 0.987 | 0.683* | 0.657 | 0.710 |
|  | Cardiology | 1.021 | 0.922 | 1.129 | 0.631* | 0.557 | 0.715 |
|  | Orthopaedics | 1.059 | 0.950 | 1.180 | 0.554* | 0.483 | 0.636 |
|  | Primary | 0.965* | 0.945 | 0.985 | 0.427* | 0.410 | 0.446 |
|  | **Overall** | **1.023*** | **1.007** | **1.039** | **0.770*** | **0.756** | **0.785** |
| Medicaid | Dermatology | 0.624 | 0.388 | 1.003 | 1.099 | 0.779 | 1.551 |
|  | Psychiatry | 0.992 | 0.981 | 1.003 | 1.004 | 0.981 | 1.027 |
|  | Endocrinology | 1.009 | 0.945 | 1.078 | 0.612* | 0.546 | 0.686 |
|  | Cardiology | 1.425* | 1.122 | 1.810 | 0.602* | 0.414 | 0.877 |
|  | Orthopaedics | 0.952 | 0.771 | 1.176 | 1.042 | 0.876 | 1.240 |
|  | Primary | 1.020 | 0.980 | 1.063 | 0.435* | 0.397 | 0.477 |
|  | **Overall** | **1.053*** | **1.021** | **1.086** | **0.729*** | **0.699** | **0.760** |
| Self-Pay | Dermatology | 0.624 | 0.260 | 1.499 | 1.319 | 0.840 | 2.071 |
|  | Psychiatry | 1.011* | 1.004 | 1.018 | 0.800* | 0.763 | 0.839 |
|  | Endocrinology | 0.849* | 0.749 | 0.961 | 0.758* | 0.649 | 0.885 |
|  | Cardiology | 1.085 | 0.761 | 1.546 | 0.960 | 0.652 | 1.413 |
|  | Orthopaedics | 0.858 | 0.648 | 1.135 | 0.445* | 0.278 | 0.713 |
|  | Primary | 1.311* | 1.255 | 1.369 | 0.586* | 0.537 | 0.638 |
|  | **Overall** | **1.290*** | **1.247** | **1.336** | **0.705*** | **0.671** | **0.741** |

RR = risk ratio, CI = confidence interval, LL = lower limit, UL = upper limit

^a^ “Overall” refers to the aggregation of the six medical specialties listed.

* = P < 0.05.

**Table B3. Relative Use of Telemedicine and Relative Use of Video Within Telemedicine by Patient Age, July 1 to September 30, 2020, by Clinical Service Line^a^**

| **vs. Ages 56-74 (Baby Boomers** |  | **Use of Telemedicine** | | | **Use of Video, Within Telemedicine Subgroup** | | |
| --- | --- | --- | --- | --- | --- | --- | --- |
|  |  | **RR** | **CI LL** | **CI UL** | **RR** | **CI LL** | **CI UL** |
| Ages 75+ (Silent, Greatest) | Dermatology | 0.769 | 0.552 | 1.072 | 0.700 | 0.480 | 1.023 |
|  | Psychiatry | 1.011* | 1.005 | 1.018 | 0.946* | 0.933 | 0.958 |
|  | Endocrinology | 1.006 | 0.960 | 1.055 | 0.692* | 0.637 | 0.752 |
|  | Cardiology | 0.964 | 0.863 | 1.076 | 0.673* | 0.564 | 0.803 |
|  | Orthopaedics | 1.139 | 0.971 | 1.337 | 0.760* | 0.598 | 0.964 |
|  | Primary | 0.992 | 0.961 | 1.023 | 0.585* | 0.542 | 0.632 |
|  | **Overall** | **1.110*** | **1.085** | **1.134** | **0.969*** | **0.942** | **0.997** |
| Ages 40-55 (Gen X) | Dermatology | 1.191 | 0.887 | 1.601 | 1.119 | 0.885 | 1.416 |
|  | Psychiatry | 1.018* | 1.012 | 1.024 | 0.986* | 0.975 | 0.997 |
|  | Endocrinology | 1.050* | 1.013 | 1.088 | 1.209* | 1.162 | 1.259 |
|  | Cardiology | 1.182* | 1.039 | 1.345 | 1.192* | 1.022 | 1.390 |
|  | Orthopaedics | 0.930 | 0.811 | 1.067 | 1.268* | 1.089 | 1.476 |
|  | Primary | 1.148* | 1.120 | 1.176 | 1.456* | 1.397 | 1.517 |
|  | **Overall** | **1.180*** | **1.158** | **1.203** | **1.207*** | **1.181** | **1.234** |
| Ages 25-39 (Millennials) | Dermatology | 1.369* | 1.000 | 1.874 | 0.834 | 0.608 | 1.143 |
|  | Psychiatry | 1.010* | 1.003 | 1.017 | 0.877* | 0.860 | 0.895 |
|  | Endocrinology | 1.118* | 1.076 | 1.163 | 1.263* | 1.211 | 1.317 |
|  | Cardiology | 1.201* | 1.008 | 1.432 | 1.377* | 1.146 | 1.655 |
|  | Orthopaedics | 1.253* | 1.074 | 1.461 | 1.554* | 1.340 | 1.803 |
|  | Primary | 1.275* | 1.241 | 1.309 | 1.701* | 1.632 | 1.774 |
|  | **Overall** | **1.329*** | **1.301** | **1.356** | **1.255*** | **1.226** | **1.284** |
| Ages 10-24 (Gen Z) | Dermatology | 1.427* | 1.031 | 1.974 | 0.990 | 0.744 | 1.317 |
|  | Psychiatry | 0.977* | 0.961 | 0.993 | 1.030* | 1.019 | 1.041 |
|  | Endocrinology | 1.006 | 0.929 | 1.090 | 1.163* | 1.069 | 1.264 |
|  | Cardiology | 1.081 | 0.756 | 1.544 | 1.011 | 0.633 | 1.617 |
|  | Orthopaedics | 1.153 | 0.985 | 1.351 | 1.740* | 1.514 | 2.000 |
|  | Primary | 0.966 | 0.926 | 1.008 | 1.714* | 1.618 | 1.816 |
|  | **Overall** | **0.908*** | **0.878** | **0.938** | **1.332*** | **1.290** | **1.375** |
| Ages 0-9 (Gen Alpha) | Dermatology | 1.033 | 0.677 | 1.578 | 1.594* | 1.318 | 1.928 |
|  | Psychiatry | 1.008 | 0.995 | 1.022 | 0.701* | 0.653 | 0.752 |
|  | Endocrinology | - | - | - | - | - | - |
|  | Cardiology | - | - | - | - | - | - |
|  | Orthopaedics | 0.860 | 0.655 | 1.128 | 2.160* | 1.859 | 2.510 |
|  | Primary | 0.639* | 0.584 | 0.699 | 1.702* | 1.522 | 1.904 |
|  | **Overall** | **0.695*** | **0.654** | **0.740** | **1.286*** | **1.213** | **1.364** |

RR = risk ratio, CI = confidence interval, LL = lower limit, UL = upper limit

^a^ “Overall” refers to the aggregation of the six medical specialties listed.

* = P < 0.05.

**Table B4. Relative Use of Telemedicine and Relative Use of Video Within Telemedicine by Patient Sex, July 1 to September 30, 2020, by Clinical Service Line^a^**

| **vs. Female** |  | **Use of Telemedicine** | | | **Use of Video, Within Telemedicine Subgroup** | | |
| --- | --- | --- | --- | --- | --- | --- | --- |
|  |  | **RR** | **CI LL** | **CI UL** | **RR** | **CI LL** | **CI UL** |
| Male | Dermatology | 0.861 | 0.700 | 1.060 | 1.186 | 0.996 | 1.413 |
|  | Psychiatry | 0.994* | 0.988 | 0.999 | 0.975* | 0.964 | 0.987 |
|  | Endocrinology | 1.064* | 1.034 | 1.096 | 1.012 | 0.976 | 1.049 |
|  | Cardiology | 0.871* | 0.795 | 0.954 | 0.980 | 0.867 | 1.109 |
|  | Orthopaedics | 1.009 | 0.914 | 1.115 | 1.031 | 0.930 | 1.143 |
|  | Primary | 0.789* | 0.773 | 0.805 | 1.100* | 1.065 | 1.136 |
|  | **Overall** | **0.779*** | **0.767** | **0.791** | **1.027*** | **1.010** | **1.045** |

RR = risk ratio, CI = confidence interval, LL = lower limit, UL = upper limit

^a^ “Overall” refers to the aggregation of the six medical specialties listed.

* = P < 0.05.
